# The neurocognitive basis of Chinese idiomatic constructions and processing differences between native speakers and L2 learners of Mandarin

**DOI:** 10.3389/fpsyg.2023.1112611

**Published:** 2023-02-23

**Authors:** Te-Hsin Liu, Chia-Ho Lai, Tai-Li Chou

**Affiliations:** ^1^Graduate Program of Teaching Chinese as a Second Language, National Taiwan University, Taipei, Taiwan; ^2^Department of Psychology, New York University, New York, NY, United States; ^3^Department of Psychology, National Taiwan University, Taipei, Taiwan; ^4^Graduate Institute of Linguistics, National Taiwan University, Taipei, Taiwan

**Keywords:** Chinese idioms, semantics, syntax, left anterior temporal lobe, anterior cingulate cortex, second language acquisition

## Abstract

Classic linguistic analyses assume that syntax is the center of linguistic system. Under this assumption, a finite set of rules can produce an infinite number of sentences. By contrast, construction grammar posits that grammar emerges from language use. Chinese quadrisyllabic idiomatic expressions (QIEs) offer a testing ground for this theoretical construct owing to their high productivity. To understand the cognitive processing of structure and meaning during reading comprehension, we used a semantic judgment task to measure behavioral performance and brain activation (functional MRI). Participants were 19 Mandarin native speakers and 19 L2 learners of intermediate and advanced levels of Mandarin. In the task, participants were instructed to indicate whether the interpretation of a QIE was correct. Our behavioral results showed that L2 learners processed high frequency QIEs faster than low frequency ones. By contrast, low frequency QIEs were processed faster than high frequency ones by native speakers. This phenomenon may be attributed to semantic satiation which impedes the interpretation of high frequency QIEs. To unravel the puzzle, a further functional MRI experiment on native speakers was conducted. The results revealed that the comparison of high-frequency and low-frequency QIEs promoted significant anterior cingulate activation. Also, the comparison of idiomatic and pseudo-idiomatic constructions exhibited significant activation in the bilateral temporal poles, a region that computes semantics rather than syntactic structure. This result indicated that, for native speakers, processing Chinese idiomatic constructions is a conceptually driven process.

## 1. Introduction

Before the emergence of language, infants refer to several pre-language behaviors, such as pointing, eye contact, and social reference, to communicate their intention. At the onset of word production, the language of infants is primarily composed of one word, which is the pairing of signified (concept) and signifier (sound), with syntax emerging at a later stage. Analyzing the neural substrates of meaning composition, neurolinguistic research has also identified “a system of composition that involves rapidly peaking activity in the left anterior temporal lobe” (Bemis and Pylkkänen, 2013; Pylkkänen, 2019a,b). This brain region is related to the shared processing between comprehension and production and computes meaning rather than syntactic structure. These findings are contradictory to the traditional componential model, in which a speaker’s grammatical knowledge is organized into syntactic, semantic, and phonological components, with all grammar above the word level explainable using highly general rules. Chinese quadrisyllabic idiomatic expressions (QIEs) offer a testing ground for this theoretical debate owing to their high productivity in the modern language. These expressions are to understand the cognitive processing of meaning and structure during reading comprehension, for example, the expression of [*qian-A-wan-B*] “1 k-A-10 k-B” (e.g., 1,000 army 10, 000 horse).

Idiomatic expressions are conventional expressions that have a meaning distinct from the meaning of the constituent words. Idioms involve metaphors (e.g., *love is fire*) and metonymies (e.g., *count heads*); consequently, the precise figure involved can be difficult to determine, such as in *kick the bucket* (Croft and Cruse, 2004). Although some studies have reported that idioms may be lexically flexible (e.g., *button one’s lip* → *fasten one’s lip*), productive (e.g.*, roll out the carpet* → *sweep up the carpet*; Gibbs et al., 1989; McGlone et al., 1994) and undergo syntactic processing (Peterson et al., 2001), other studies have determined idioms to be noncompositional (Swinney and Cutler, 1979) and processed formally, as is the case with single words (Kempler et al., 1999).

Because many Chinese QIEs originate from well-known passages in classic works, they are highly synthetic and concise.^1^ By contrast, the syntactic structure of idiomatic expressions in European languages is similar to regular phrase structures, such that they can undergo syntactic operations including passive voicing, insertion, topicalization, and quantification (Cacciari and Glucksberg, 1994; Cacciari, 2014). For instance, the idiom *kick the bucket* and the phrase *kick the ball* share the same grammatical structure; furthermore, most native English speakers would accept the passive version of the idiom *pop the question* (Gibbs et al., 1989). However, Chinese idioms rarely allow change in the syntactic structure (Liu et al., 2019).

The psychological basis of idiom processing has been investigated in depth over the last three decades, with the focus directed toward semantic compositionality as well as the role of frequency in idiom processing. The Principle of Semantic Compositionality, sometimes called “Frege’s Principle,” is based on the assumption that the whole meaning of a sentence can be reconstructed by means of its component parts (Nunberg et al., 1994; Pelletier, 1994). Compositionality means that the grammar obeys the “rule-to-rule hypothesis,” such that the meaning of the whole can be inferred from the meaning of its parts. A challenge for compositionality has been observed in the interpretation of idioms. For instance, in the sentence “Peter *made no bones about* wanting to be promoted,” the literal meaning of the italicized phrase is not relevant to the understanding of the sentence, and the meaning of the whole cannot be computed in a compositional sense.

Early theories of idiom comprehension relied on the assumption that a literal interpretation has priority over a figurative meaning, and the assumption was rejected only when the figurative meaning was activated (Bobrow and Bell, 1973). This line of thinking indicates the criticality of semantic transparency in idiom processing.

Taking the role of frequency into account, Cacciari and Tabossi (1988) developed the Configuration Hypothesis, arguing that idioms constitute complex arrangements of single words. An essential claim of this model is that every idiom contains one or more “keys.” An idiom key acts as a type of mental signal, evoking the idiomatic configuration holistically for the hearer, which results in the activation of the idiomatic meaning. The hearer first interprets the semantics of incoming word strings based on the literal meaning, but the figurative meaning of the idiom is activated as long as adequate information is collected. Moreover, the key to the rapid retrieval of idiomatic expressions is frequency, with the major difference between idiomatic and literal expressions being that speakers and listeners are familiar with idiomatic expressions, whereas literal expressions are entirely novel. Their semantic judgment paradigm showed that there was no significant difference between decomposable and nondecomposable idioms, suggesting that the role of semantic transparency is insignificant in idiom processing. Contrarily, the high frequency of an idiom accounts for its rapid recognition. Titone and Connine (1999) advocated for a hybrid model of idiom comprehension characterizing idioms both as unitary word configurations and compositional word sequences. Based on this model, both the literal and the figurative meanings were activated. Their results of an eye-tracking experiment demonstrated that reading rates differ as a function of the inherent decompositionality of idioms. It took longer for non-compositional idioms to integrate correct meaning into the idiomatic context given that two meanings were semantically distinct.

**Figure 1:**
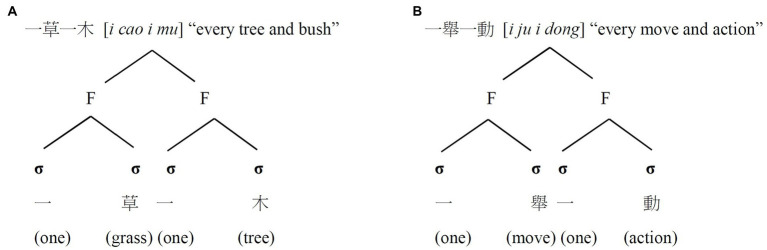
Illustration of the internal morphology of two QIEs with the same syntactic structure.

The frequency examined by Cacciari and Tabossi (1988) and Titone and Connine (1999) refers to an idiom’s “token frequency” rather than its “type frequency.” Token frequency reflects the total number of words in the input, and type frequency represents the extent of use or realized productivity of a certain pattern. The productivity of linguistic patterns is reflected through type frequency rather than token frequency (Bybee and Hopper, 2001). This insight is crucial in relation to Chinese idiomatic expression, many of which are highly productive and thus have a high type frequency. For instance, 49 idiomatic expressions have the structure [*i-A-i-B*] “one-A-one-B” (Liu et al., 2019), reflecting a fixed construction and high productivity. Figure 1 illustrates the two idioms with the coordinate form, in which the first and second foot are semantically and structurally equivalent.

The restricted possibilities for the internal morphological and syntactic structures of Chinese QIEs can be analyzed by Construction Grammar (CG) which claims that constructions are the essential building blocks of human language (Fillmore et al., 1988; Goldberg, 1995). Goldberg defined a construction as “the pairing of form and meaning.” For example, for *The bigger they come, the harder they fall*, proponents of CG do not reduce this proverb to a mere fixed phrase but identify a template (*The Xer, the Yer*) with “slots” that can be filled with almost any comparative phrase (e.g., *The more you want, the less you get*). Proponents of CG claim that such idiosyncratic templates are frequent in the language and that they can be understood as multiword, partially filled constructions. Construction being the pairing of form and meaning, semantic compositionality is thus derived from the interaction between the meaning of a construction and the slots filled in the construction.

Based on Construction Grammar, Liu et al. (2019) suggested that one of the meaning of the prefab [*i-A-i-B*] “one-A-one-B” is “each and every.” For instance, 一草一木 [one-grass-one-tree] means “every tree and bush” and 一舉一動 [one-move-one-action] means “every movement and every action.”

Pursuing the same line of research, Liu and Su (2021) compared the difference between Mandarin speakers and advanced learners of Mandarin in the processing of Chinese idiomatic constructions. In their experiment, 23 Mandarin speakers and 23 advanced learners of the non-Sinosphere were instructed to make semantic judgments in response to a set of QIEs. The results indicated that both the L1 group and the L2 group processed semantically transparent QIEs quicker than semantically opaque ones. The native speakers also processed low-frequency QIEs quicker than high-frequency QIEs, suggesting that semantic satiation plays a role in impeding the recognition of high-frequency idioms. A question arises as to the neural regions involved during the processing of idiomatic constructions and whether neural correlates are dominated by syntax or semantics. Meanwhile, the divergence between native speakers and L2 learners in the processing of idiomatic expressions deserves further investigation.

Arguing for the independence of syntax from semantics, classic linguistic theories place syntax at the core of language theory. Syntax has been recognized as the focal point of language production, with semantics and morphophonology representing interpretations of syntactic structures through a series of transformational rules. Research has addressed this theoretical topic, demonstrating that neither comprehension nor production is syntactic and that the left anterior temporal lobe (ATL) has a distinctly conceptual profile. Westerlund and Pylkkänen (2014) manipulated the specificity of object concepts, and analyzed the brain responses of participants processing combinations with low-specificity nouns (e.g., blue boat) or with high-specificity counterparts (e.g., blue canoe). Frequency and the transition probability between adjective and noun were carefully controlled. Their result revealed that the left ATL responded more strongly (250 ms after noun presentation) for low-specificity combinations than for high-specificity combinations, indicating that the left ATL is associated with the combinatorial aspects of the noun, such as conceptual specificity. Subsequent studies have also identified a system of composition that involves rapidly peaking activity in the left ATL followed by later engagement of the medial prefrontal cortex. Both brain regions are related to the shared processing between comprehension and production and compute meaning rather than syntactic structure (Pylkkänen, 2019a,b). Bemis and Pylkkänen (2013) and Flick et al. (2018) determined that, insofar as the task is to combine meanings, the left ATL can operate without local syntactic combinations. Thus, in the processing of natural language, neural signals are dominated by correlates of semantics rather than syntax.

Two neurolinguistic studies have investigated the processing of Chinese QIEs. Zhang et al. (2013) used event-related potentials (ERPs) to investigate the role of semantic compositionality during the interpretation of idioms. The researchers visually presented 146 QIEs of varying compositionality and nonidiomatic phrases to 18 participants as a semantic judgment task. Their behavioral results demonstrated that, in Mandarin, the meaning of individual characters remained active during the comprehension of idioms. In addition, literal nonidiomatic phrases elicited longer reaction times (RTs) compared with all the Chinese idioms. Consequently, their result demonstrated that the degrees of semantic compositionality may affect the activation of figurative meaning. Investigating the role of the right hemisphere in Chinese idiom processing, Yang et al. (2016) determined that both the left and right hemispheres contribute to the recognition of idiomatic expressions but through different pathways. Both opaque and transparent idioms elicited more activation than nonidioms in the right superior parietal lobule as well as right precuneus. Meanwhile, the activation strength was negatively correlated with the semantic transparency of the idioms. Their result contradicted the graded salience hypothesis (Giora, 1997, 2003), which predicts that the processing of familiar phrases mainly involves the left hemisphere and not the right hemisphere.

In summary, semantic transparency and frequency were both determined to play a role in Chinese idiom processing. However, which neural regions are involved in the processing of idiomatic constructions remains unclear. Do speakers prioritize meaning or syntactic structure? What is the difference between native speakers and L2 learners in the processing of idiomatic expressions? It has been shown that frequency, semantic transparency, familiarity as well as L1-L2 similarity are important factors in L2 idiom processing (Steinel et al., 2007; Garcia et al., 2015). Many Chinese idioms are highly productive and can be analyzed from a constructional perspective. The neural regions involved in the processing of idiomatic constructions can elucidate whether the neural correlates are dominated by syntax or semantics. Moreover, how construction interacts with frequency and the processing difference between native speakers and L2 learners warrant further investigation. The study of Chinese idioms, from a constructional perspective, is crucial on account of the syntactic/semantic parallelism as well as productivity of these idioms, but no fMRI-based research on this topic has been conducted to date.

To bridge the research gaps, the construction [*qian-A-wan-B*] “1 k-A-10 k-B” was selected given that it is composed of 50 idiomatic sequences. Highly productive but varying in token frequency, [*qian-A-wan-B*] “1 k-A-10 k-B” is suitable for investigating the respective role of frequency and construction. In terms of syntax, the A and B of [*qian-A-wan-B*] “1 k-A-10 k-B” can be nearly synonymous nouns, as in [*qian-chou-wan-hen*] (千仇萬恨: [1 K-hate-10 K-resentment] “deep hatred”). They can also be semibound morphemes that form a disyllabic verb, as in [*qian-ding-wan-zhu*] (千叮萬囑: [1 K-urge-10 K-advise] “exhort repeatedly”). The construction [*qian-A-wan-B*] “1 k-A-10 k-B” is symmetrical given that *qian* “1 k” and *wan* “10 k” are numerals and that A and B have equivalent syntactic function. In terms of semantics, *qian* “1 k” and *wan* “10 k” both mean “large in amount.” Combined with the above expressions, the construction emphasizes the action to execute on the amount of a noun. Meanwhile, given that the construction is the pairing of form and meaning, the comprehension of idiomatic constructions does not rely on the context in which they occur; instead, the construction itself is the “context” where the native speaker relies on to interpret the meaning.

## 2. Materials and methods

### 2.1. Participants

Nineteen university-level Mandarin speakers (age = 24.1 ± 3 years old, age range 20–28 years) and 19 L2 learners of intermediate and advanced levels of Mandarin (age = 24.6 ± 3.4 years old, age range 20–32 years) took part in this study. The L2 participants were all native speakers of the non-Sinosphere.^2^ All participants, right-handed, had normal or corrected-to-normal vision and had no language disorder. The study was approved by the Research Ethics Committee of our university, and all the participants provided their informed consent before participating in the experiment.

The revised version of Peabody Picture Vocabulary Test (PPVT) was administered to estimate L2 subjects’ receptive lexicon (Sun et al., 2022). The L2 participants’ mean score was 83.45, indicating that they had the advanced level of reading proficiency in Mandarin.

### 2.2. Stimuli

To obtain the frequency of idioms, the Center for Chinese Linguistics (CCL; Beijing University) Corpus containing 4.77 hundred million characters was employed at the first stage. However, seven of the 48 existing constructions were not available in the CCL corpus. We then referred to Google Search to obtain up-to-date frequencies. We acknowledge that frequency information gathered using search engines must be treated carefully; however, such information can reflect the frequency with which certain words and syntactic patterns occur (Meyer et al., 2001). We therefore individually entered the 48 [*qian-A-wan-B*] “1 k-A-10 k-B” idioms into Google Search to obtain the latest frequency information.

The experimental stimuli included the following: (1) 48 [*qian-A-wan-B*] “1 k-A-10 k-B” idiomatic constructions divided by frequency; (2) 16 pseudo [*qian-A-wan-B*] “1 k-A-10 k-B” trials violating the semantic constraint of the construction^3^; and (3) 16 non-constructional idioms, such as 龍飛鳳舞 (*long-fei-feng-wu*, “lively and vigorous in calligraphy”) and 虎頭蛇尾 (*hu-tou-she-wei*, “fine start and poor finish”). A summary of the experimental stimuli is presented in Table 1. Because pseudo-idiomatic constructions do not exist in Mandarin, we can only provide word-by-word meanings. A *t* test assessed that a reliable difference in frequency existed between high-frequency and low-frequency idiomatic constructions [*t* (23) = 4.73, *p* < 0.001]. The linguistic material is presented in the Appendix.

**Table 1:** Examples of trials in the experiment.

Condition	Example	Meaning	Mean frequency	No. of items
*Idiomatic constructions*				
High Frequency	千軍萬馬 *(qian-jun-wan-ma)*	A huge army	199,231	24
Low Frequency	千生萬死 *(qian-sheng-wan-si)*	Vary dangerous situation	1,764	24
*Pseudo-idiomatic constructions*	千竹萬酒 *(qian-zhu-wan-jiu)*	[*1 k-bamboo-10 k-wine*]	0.46	16
*Not construction-based idioms*	龍飛鳳舞 *(long-fei-feng-wu)*	Lively in calligraphy	15,252,792	16

### 2.3. Experimental paradigm

**Figure 2:**
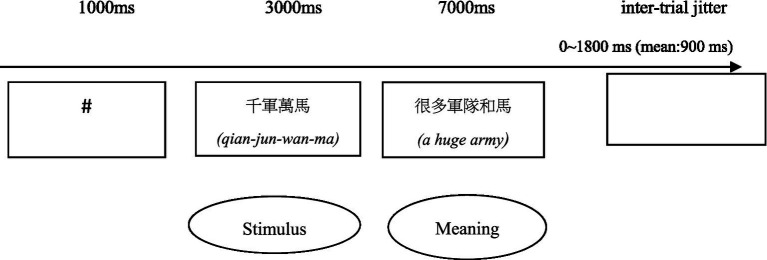
Illustration of the experimental procedure.

The present study used an event-related fMRI paradigm consisting of four runs. Each run had an experimental block and a baseline block. The experimental block contained 12 idiomatic constructions (divided on the basis of frequency) and four pseudo-idiomatic constructions. The baseline block consisted of four idioms that were not construction-based. All idioms were presented randomly. Each trial began with a fixation cross centered on the screen, which was displayed for 1,000 ms. Subsequently, each participant was presented with 80 stimuli. Each stimulus was displayed for 3,000 ms in random order and was followed by its meaning. The participants had at most 5 s to decide whether the displayed meaning was correct or incorrect. Before the formal test, 10 prime-target trials that were not used in the study were administrated for practice. All participants familiarized themselves with the task through this procedure. The experiment lasted approximately 15 min. The pseudo-idiomatic constructions were all conceived, and all answers in this category were thus incorrect. Before getting into the fMRI scanner, participants were reminded to keep still (Figure 2).

### 2.4. Image acquisition

Images were captured using a Siemens 3 T Prisma scanner with a 20-channel head coil at our university. Participants were in the scanner with their head position fixed. The head coil was placed around the participants’ heads. A response box was held in the participants’ right hands, and the visual materials were given to them through a goggle. Gradient-echo localizer images were used to decide the position of the functional slices. For functional images, a susceptibility-weighted single-shot echo-planar imaging (EPI) method to measure blood oxygen was used. Functional images were obtained in parallel to the AC–PC plane with interleaved EPI acquisition in the whole brain from bottom to top. The scanning parameters were as follows: TR = 2,000 ms, TE = 24 ms, flip angle = 90°, matrix size = 64 × 64, field of view = 192, slice thickness = 3.5 mm, and number of slices = 36. All participants completed four functional runs with 143 image volumes for each run (4.8 min/run, total time: 19.2 min). A high-resolution, T1-weighted 3D image was used with the following parameters: TR = 2,000 ms, TE = 2.3 ms, flip angle = 8°, matrix size = 256 × 256, field of view = 240, slice thickness = 0.94 mm, and number of slices = 192.

### 2.5. Image analysis

Images were preprocessed using Statistical Parametric Mapping software (SPM8, Wellcome Department of Cognitive Neurology, London, United Kingdom). Then, we used the middle slice volume to correct for functional images collected in different slice acquisition time. Functional images were realigned to the first volume in each session *via* affine transformations. No subject had more than 3 mm of movement in any plane. Co-registered images were normalized to the Montreal Neurological Institute space (voxel size: 3 × 3 × 3 mm^3^). We used smoothed data for statistical analyses with a 10-mm full-width-at-half-maximum Gaussian kernel. A high-pass filter (128-s cutoff period) was applied to minimize low-frequency artifacts. For whole brain analysis, statistical analysis relied on the general linear model by using event-related analysis. Materials in the three conditions were treated as events and were modeled for the idiomatic meaning using a canonical hemodynamic response function (HRF). There were three event types: idiomatic constructions, pseudo-idiomatic constructions, and non-constructional idioms. Parameter estimates from the canonical HRF contrasts were entered into random-effects models in single-subject models by using single-sample *t* tests across participants in a whole-brain analysis. We compared idiomatic constructions to pseudo-idiomatic constructions. Among idiomatic constructions, we also compared high-frequency and low-frequency idioms. Studies have reported that the left anterior temporal lobe (or ATL, BA 38) is a critical region for compositional processing (Bemis and Pylkkänen, 2013; Pylkkänen, 2019a,b). Considering the involvement of the right hemisphere in idiom processing, we used the bilateral ATL mask from the AAL atlas for the (idiomatic constructions vs. pseudo-idiomatic constructions) contrast. In addition, Benedek et al. (2014) revealed that the dorsal posterior cingulate cortex is associated with figurative language processing. Thus, we used the bilateral midcingulum mask (dorsal part of the cingulate cortex) from the AAL atlas for (high-frequency vs. low-frequency) contrast. All the reported brain regions listed in Table 2 were FDR-corrected (*p* < 0.05) with the use the aforementioned masks as regions of interest (ROI) analysis.

**Table 2:** fMRI results in the whole-brain analyses.

Regions	H	BA	voxels	*z* value	MNI coordinates		
					*x*	*y*	*z*
High-frequency > Low-frequency							
Mid-Cingulate Cortex (dorsal Anterior Cingulate Cortex)	I	32	64	3.29	0	11	38
Mid-Cingulate Cortex (dorsal Posterior Cingulate Cortex)	I	31	102	3.41	0	−31	38
							
Idiomatic > Pseudo-idiomatic							
Temporal Pole	L	38	27	3.18	−57	5	−5
Temporal Pole	R	38	44	3.81	60	5	−5

The reported areas were FDR-corrected (*p* < 0.05) at the peak level by using masks from the AAL atlas. H, hemisphere; I, interhemispheric; L, left; R, right; and BA, brodmann area.

## 3. Analysis

### 3.1. Study 1: Behavioral results of native speakers and L2 learners

**Figure 3:**
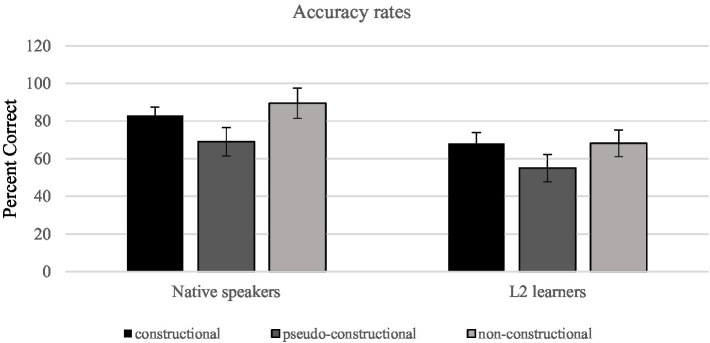
Accuracy rates for L1 group and L2 group. The error bars represent SDs.

In the L1 group, the accuracies (mean) for the constructional, pseudo-constructional, and non-constructional conditions were 83, 61, and 90%. In the L2 group, the accuracies (mean) for the constructional, pseudo-constructional, and non-constructional conditions were 68, 55, and 67%. A 2 group (L1, L2) × 3 condition (constructional, pseudo-constructional, and non-constructional) ANOVA on accuracy was performed. Mauchly’s test of sphericity indicated that sphericity could not be assumed [*χ^2^*(2) = 13.473, *p* = 0.001]. To deal with violations of sphericity, we used Greenhouse–Geisser estimates of sphericity to correct the degrees of freedom. The effect of construction type was significant [*F* (1.5, 54.6) = 27.3, *p* < 0.001, *η^2^* = 0.43]. Meanwhile, the ANOVA also revealed a significant main effect for language background. The L1 group obtained higher accuracy rates (77.79 vs. 63.71%). Meanwhile, pairwise comparisons showed a significant difference between L1 group and L2 group [*F* (1, 36) = 62.4, *p* < 0.001, *η^2^* = 0.63]. The interaction between construction type and language was also significant [*F* (1.5, 54.6) = 3.78, *p* = 0.04, *η^2^* = 0.095]. It can be observed that different types of construction led to distinct behavioral outcomes, and that the L1 group obtained better results on all types of idioms compared with the L2 group, as can be seen in Figure 3.

Further pairwise comparisons on construction type for each group of subjects were conducted. A Bonferroni correction adjusted the alpha level for the three comparisons to 0.017. Overall, there was no significant difference in accuracy rates between constructional and non-constructional idioms for both groups. Nevertheless, the accuracies between constructional and pseudo-constructional idioms were significant for native speakers (*p* = 0.003) but not significant for L2 learners, indicating that L2 learners were not aware of the agrammaticalness of pseudo-constructional idioms.

To explore how frequency and language background interact, we then conducted a two-way ANOVA to analyze the accuracy rates for constructional idiom (between-subject variable: language background; within-subject variable: frequency). The frequency × language interaction was significant [*F* (1, 36) = 5.40, *p* = 0.026, *η^2^* = 0.130]. Meanwhile, the statistical result also revealed a significant main effect for language background. The L1 group obtained higher accuracy rates (L1: 83.00% vs. L2: 68.42%). Pairwise comparisons also revealed a significant difference between L1 group and L2 group [*F* (1, 36) = 17.47, *p* < 0.001, *η^2^* = 0.327]. The effect of frequency was not significant [frequency: *F* (1, 36) = 1.667, *p* = 0.205]. Meanwhile, contrary to native speakers, L2 learners gained higher accuracy rates in high-frequency idioms (high-frequency idioms: 69.51% vs. low-frequency idioms: 67.32%).

**Figure 4:**
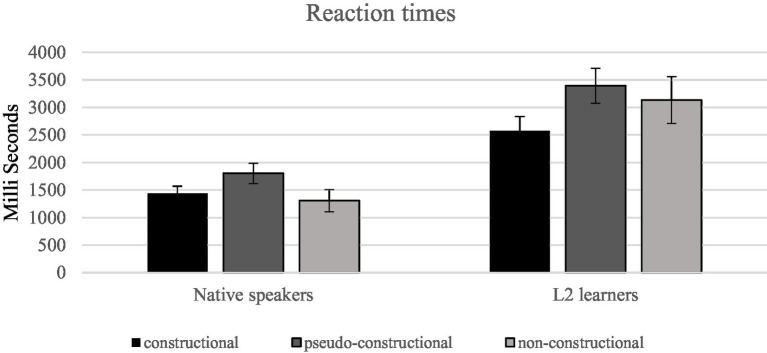
RTs for L1 group and L2 group by construction type. The error bars represent SDs.

The mean RTs for the constructional, pseudo-constructional, and non-constructional conditions were 1,442, 1,803, and 1,306 ms in the L1 group. The mean RTs for the constructional, pseudo-constructional, and non-constructional conditions were 2,578, 3,394, and 3,126 ms in the L2 group. A 2 group (L1, L2) × 3 condition (constructional, pseudo-constructional, and non-constructional) ANOVA on RTs was performed. The assumption of sphericity had been violated [*χ^2^*(2) = 11.547, *p* = 0.003], therefore, we used Greenhouse–Geisser estimates of sphericity to correct degrees of freedom. The effect of construction type was significant [*F* (1.56, 56.21) = 49.92, *p* < 0.001, *η^2^* = 0.581]. Meanwhile, the statistical result also revealed a significant main effect for language background. The L1 group took shorter time to respond than the L2 group (L1: 1,258 ms. vs. L2: 2,805 ms.). Pairwise comparisons revealed a significant difference between L1 group and L2 group [*F* (1, 36) = 95.16, *p* < 0.001, *η^2^* = 0.726]. The construction type × language interaction was also significant. [*F* (1.56, 56.21) = 10.74, *p* < 0.001, *η^2^* = 0.23]. Further pairwise comparisons on construction type for each group of subjects were conducted. A Bonferroni correction adjusted the alpha level for the three comparisons to 0.017. For both group of speakers, the difference in RTs between constructional and pseudo-constructional idioms was significant, with the former taking shorter time to respond. Meanwhile, for the L2 group, no significant difference in RTs was observed between non-constructional and pseudo-constructional idioms. For the L1 group, no significant difference in RTs was observed between constructional and non-constructional idioms (see Figure 4).

We further conducted a two-way ANOVA to analyze the RTs for constructional idioms (between-subject variable: language background; within-subject variable: frequency). The frequency × language interaction was significant [*F* (1, 36) = 5.53, *p* = 0.024, *η^2^* = 0.133]. Moreover, the statistical result revealed a significant main effect for language background. The L2 group had slower responses compared with native speakers (native: 1,364 ms. vs. L2 learners: 2,586 ms). Pairwise comparisons revealed a significant difference between L1 group and L2 group [*F* (1, 36) = 86.82, *p* < 0.001, *η^2^* = 0.707]. Nevertheless, the effect of frequency was not significant [frequency: *F* (1, 36) = 0.634, *p* = 0.431]. Meanwhile, contrary to native speakers, L2 learners gained slower responses in low-frequency idioms (high-frequency idioms: 2,498 ms vs. low-frequency idioms: 2,675 ms).

### 3.2. Study 2: fMRI results of native speakers

#### 3.2.1. High-frequency versus low-frequency idiomatic constructions

Our behavioral results demonstrated that native speakers displayed a higher accuracy rate and shorter RTs when processing idiomatic constructions with low-frequency, implying that semantic satiation (Jakobovits, 1962) impedes the interpretation of high-frequency idioms. Semantic satiation is characterized by the attenuation of meaning after uninterrupted repetition of a word (e.g., Severance and Washburn, 1907; Bassett et al., 1919). Similar effects can be observed in cases of sensory habituation, whereby repeated perceptual stimuli fade or are transformed (Warren, 1968; Kohn, 2007). Various studies have further supported the effects of semantic satiation (Balota and Black, 1997; Black, 2001). For example, Balota and Black (1997) and Kuhl and Anderson (2011) observed that, in a decision task on semantic relatedness, weakly-related words may be more satiated following repetition than highly-related words. After the repeated priming (2, 12, or 22 times) of the stimulus, participants were visually presented a word pair and were asked to evaluate its semantic relatedness; when the prime word was repeated 22 times, the relatedness effect was diminished.

**Figure 5:**
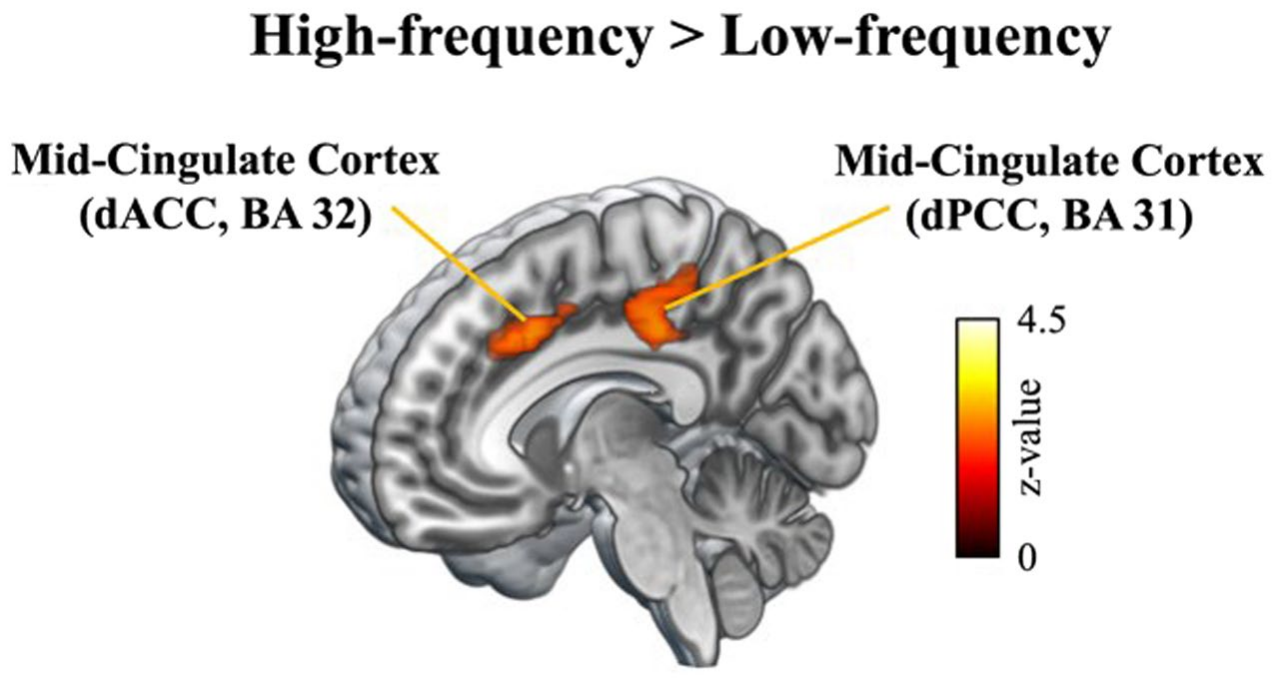
Contrast of high-frequency vs. low-frequency idiomatic constructions.

Our fMRI results demonstrated that the comparison of high-frequency and low-frequency QIEs promoted significant mid-cingulate activation (dorsal part of the anterior and posterior cingulate), suggesting that inhibitory control suppresses lasting semantic interference with high-frequency QIEs. Moreover, research has reported greater activation in the anterior cingulate cortex (ACC) in patients with obsessive–compulsive disorder and normalization of the region with treatment-induced symptom reduction (Machlin et al., 1991; Swedo et al., 1992; Perani et al., 1995; Rauch et al., 1998; Fitzgerald et al., 2005; Figure 5).

**Figure 6:**
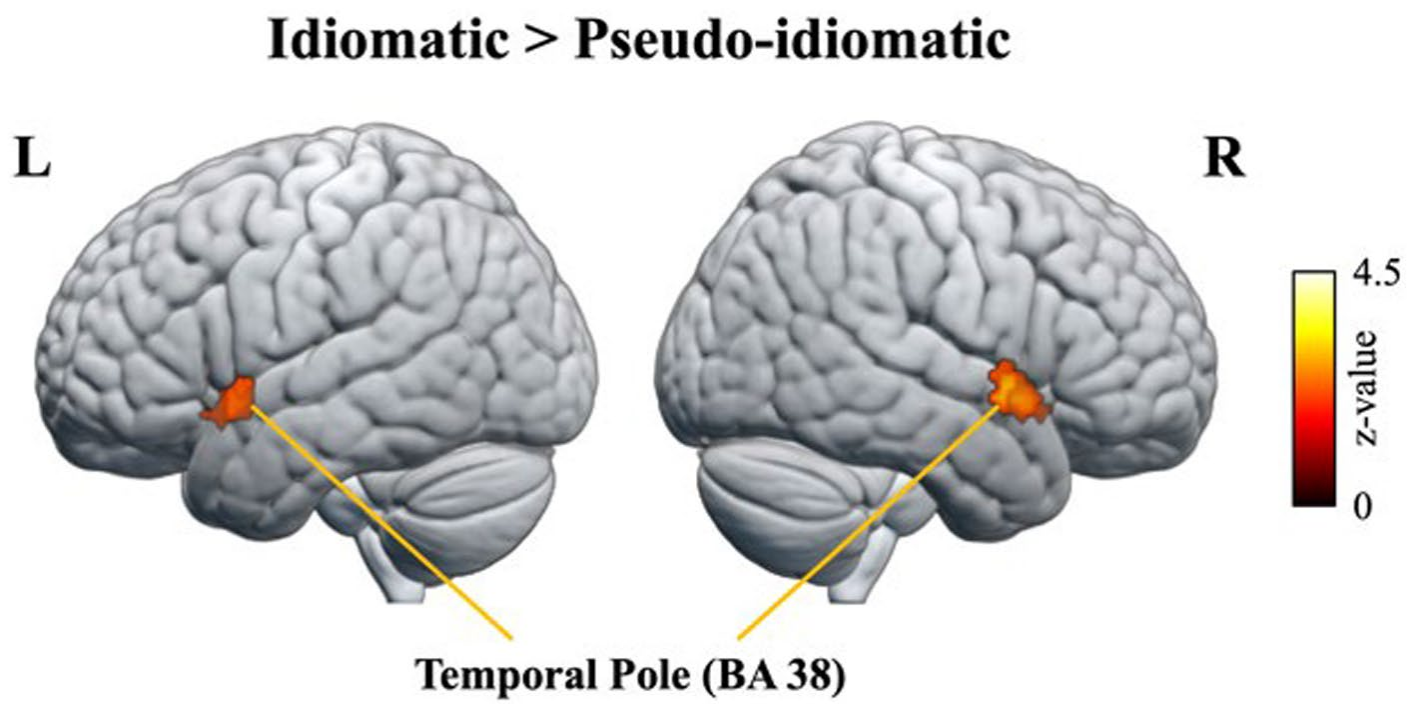
Contrast of idiomatic vs. pseudo-idiomatic constructions.

In terms of the contrast of idiomatic versus pseudo-idiomatic constructions, the participants exhibited greater activation in the bilateral ATL (BA 38), as depicted in Figure 6. It should be noted that more anterior-ventral ATL activation was found with the threshold *p* < 0.01 uncorrected in a whole brain analysis (also see a review by Patterson et al., 2007).

Rogalsky and Hickok (2009) examined task effects in the ATL regions, and found that some of which were activated only for the semantic task, some only for the syntactic task, and some for both tasks. Using rTMS in normal participants, Pobric et al. (2007) observed that the anterior temporal lobes are a critical substrate for semantic representation. Drawing upon recent neuropsychological evidence, and more specifically the striking disorder called semantic dementia (SD), Ralph et al. (2017) noted that bilateral anterior temporal lobe regions might be important for all conceptual domains, given that individuals with SD have semantic impairments across all modalities. In our data, given that the bilateral temporal lobe was activated, we thus infer that these regions compute semantic information.

## 4. Discussion

The objective of this study was 3-fold. First, we aimed to compare how native speakers and L2 learners processed different types of constructions. Second, given the intriguing behavioral result observed among native speakers, we further analyzed the neural correlates involved in the processing of Chinese idiomatic constructions. Many Chinese idioms are highly productive in the modern language and can be analyzed from a constructional perspective; consequently, the result of the study can be used to elucidate whether their processing is syntactically or conceptually driven. Third, owing to the high productivity of Chinese idiomatic constructions in the modern language, we investigated the role of frequency as well as that of construction to determine how they interact with each other.

Our behavioral results indicated that native speakers and L2 learners displayed different behaviors under the effect of frequency. The L1 group exhibited a higher accuracy rate and shorter RTs when processing idiomatic constructions with low-frequency, implying that semantic satiation (Jakobovits, 1962) impedes the interpretation of high-frequency idioms. Research has demonstrated that semantic satiation slows word associations (Balota and Black, 1997; Black, 2001). Following the assumptions of CG, the construction of idioms is the pairing of form and meaning; consequently, when processing a low-frequency idiom, participants relied on the semantics communicated by the construction to decode its meaning. In other words, intervention of a construction with direct association between form and meaning accelerates its interpretation. Nevertheless, the processing of high-frequency idioms was influenced by semantic satiation, which impeded the participants’ recognition. The effect of frequency is different for L2 learners: high-frequency idiomatic constructions had better accuracy rates than low-frequency ones. The result of RTs echoed the result for accuracy rates, with high-frequency idiomatic constructions eliciting shorter RTs than low-frequency idioms for L2 learners. This result indicated that native speakers were aware of the pairing of form and meaning conveyed by the construction, while this linguistic knowledge was absent among L2 learners.

Our fMRI results for native speakers echoed the behavioral results. The comparison of high-frequency and low-frequency QIEs elicited significant anterior cingulate activation, implying that inhibitory control suppresses lasting semantic interference in relation to high-frequency QIEs. Inhibitory control is generally related to the suppression of responses to distracting stimuli (Nigg, 2000; Friedman and Miyake, 2004; Enticott et al., 2006). It has been observed that one of the brain regions heavily involved in attention as well as inhibitory control is the ACC (Posner and Petersen, 1990; Menon et al., 2001; Garavan et al., 2002; Reischies et al., 2005). Subsequent imaging studies have reported increased ACC activation during attention tasks (Devinsky et al., 1995; Cabeza and Nyberg, 1997; Elliott and Dolan, 1998) as well as response monitoring (Taylor et al., 2007). Research has also revealed that the ACC is active among bilinguals, who use this region more efficiently to monitor cognitive conflicts beyond the linguistic domain (Abutalebi et al., 2012).

Regarding the locus of the semantic satiation effect, several ERP studies have demonstrated the role of the N400 during the repetition of a given word. Kounios et al. (2000) measured ERPs in a semantic detection task which involved repeated presentations of primes and related and unrelated words using visual and auditory stimuli. Prime satiation and prime relatedness to the key word showed interaction on the ERP amplitude to key words within the N400 time window. Ströberg et al. (2017) used a 64-channel EEG system to analyze the N400 in a semantic priming task where participants were exposed to primes repeated three or 30 times. It was observed that the N400 was reduced after 30 repetitions (versus three repetitions) for the centrofrontal electrodes. Moreover, after 30 repetitions, explorative source reconstructions suggested reduced activation in wide-spread areas. These above areas and those involved in the N400 overlap, supporting the semantic rather than the perceptual nature of the satiation effect. Our fMRI studies also demonstrated that the ACC, the area dealing with inhibitory control, attention, and response monitoring, was activated during the presentation of high-frequency idiomatic constructions. Greater activation in the ACC may act as the locus of the semantic satiation effect.

Another crucial question probed in this study was the neural basis of syntax and semantics. Our result revealed that, in terms of the contrast of idiomatic versus pseudo-idiomatic constructions, the participants exhibited greater activation in the bilateral ATL (BA 38). This finding is consistent with that of Pallier et al. (2011), namely that the ATL is associated with a constituent size effect in brain activity in the presence of lexicosemantic information, suggesting that this region encodes semantic constituents. The studies of Pylkkänen (2019a,b) have also demonstrated that the ATL computes semantics rather than syntactic structure.

Patterson et al. (2007) argued that semantic generalization depends upon a distinct amodal hub located in the ATL. Crucial to semantic memory, the ATL regions are contiguous to the anterior parts of the medial temporal lobe memory system, which manages the acquisition of new semantic information. Because episodes contribute to the progressive acquisition of new conceptual knowledge, the close proximity of the episodic and semantic systems is logical. Ralph et al. (2017) reported that the ATL semantic region exhibits positive activation for semantic tasks and deactivation for nonsemantic tasks and that individuals with semantic dementia always have bilateral ATL atrophy. Guo et al. (2013) used resting-state fMRI to determine that, in healthy participants, the ATL regions exhibit intrinsic connectivity with modality-specific brain areas. It was observed that, in patients with semantic dementia, the accuracy of comprehension was correlated with the severity of ATL atrophy as well as the degree of reduced functional connectivity of hub–spoke. The aforementioned studies have deepened our understanding of the neural bases of semantic cognition. In the present study, in terms of the contrast in idiomatic versus pseudo-idiomatic constructions, the participants exhibited greater activation in the bilateral ATL (BA 38), a region that computes semantics rather than syntactic structure (Pylkkänen, 2019a,b). This result suggests that the processing of Chinese idiomatic constructions is a conceptually driven process.

Our fMRI results have theoretical implications for linguistic theory. Classic linguistic analyses suggest that languages can be studied independently of their semantic and discourse functions and that meaning is derived from a mental dictionary of words. In the tradition of generative grammar, syntax is regarded as the center of the linguistic system, with all grammar beyond the word level explainable using highly general rules (Chomsky, 2020). Using fMRI to search for the neural mechanisms of this theoretical construct, we determined that the processing of Chinese idiomatic constructions is conceptually driven, which is consistent with the CG perspective. Under this framework, construction is viewed as “the pairing of form and meaning,” which is the essential foundation of language construction. Grammar emerges from language use, determined through usage patterns and frequency of use (Bybee and Hopper, 2001).

The present research did not deal with the processing of heritage language (HL) learners, but it has been shown that HL learners are more accurate than L2 learners in linguistic tasks such as sentence conjunction judgment task (Montrul et al., 2008). HL learners are also more target-like than L2 learners in the acquisition of grammatical aspect (Montrul, 2010). The interpretation of Chinese QIEs requires awareness of the conceptual metaphors underlying linguistic expressions as well as understanding of Chinese cultural tradition. Given that HL speakers grow up in a bilingual and bicultural environment, additional research is needed to probe how HL speakers interpret conflicting metaphors. For instance, “wine” has a positive connotation in English (e.g., *Old friends and old wine are best*), while it has a negative connotation in Mandarin (e.g., [jiu-chi-rou-lin] lit. “lakes of wine and forests of meat”; fig. “sumptuous entertainment”).

## 5. Conclusion

Primed using three types of stimuli divided on the basis of frequency and construction type, native Mandarin speakers were recruited to participate in an fMRI experiment. The results revealed that the L1 group processed low-frequency QIEs quicker than high-frequency QIEs. This result may be attributed to semantic satiation which impedes the processing of high-frequency idioms. This study provides an argument in support of CG, namely that the meaning of the construction contributes to the processing of idiomatic expressions with low-frequency.

Our fMRI results also demonstrated that the comparison of high-frequency and low-frequency QIEs elicited significant ACC activation. Furthermore, the contrast of idiomatic and pseudo-idiomatic constructions elicited greater activation in the bilateral ATL, a region that computes semantics rather than syntactic structure. This finding suggests that the processing of Chinese idiomatic constructions is a conceptually driven process.

Our results reveal several avenues that could be explored in future research on foreign language acquisition. For example, the neural correlates of frequency on idiomatic constructions among L2 learners remain unclear. Meanwhile, L2 learners’ metalinguistic knowledge and how this knowledge is mapped onto the comprehension of idiomatic constructions warrants further investigation. Finally, while the processing of Chinese idiomatic constructions is a conceptually driven process, further analysis needs to be conducted to explore whether L2 learners exhibit comparable processing mechanism.

## Data availability statement

Original contributions presented in the study are included in the article/supplementary material, further inquiries can be directed to the corresponding author.

## Ethics statement

The studies involving human participants were reviewed and approved by Research Ethics Committee, National Taiwan University. The patients/participants provided their written informed consent to participate in this study.

## Author contributions

T-HL took part in data collection, wrote the manuscript, and analyzed the behavioral data. T-HL and T-LC designed the study and provided guidance for data analysis and manuscript writing. C-HL analyzed the imaging data. All authors contributed to the article and approved the submitted version.

## Funding

This research is part of the project granted by the Ministry of Science and Technology, Taiwan (Project number: 105-2420-H-002-008-MY2) granted to the T-HL and (Project number: 105-2420-H-002-007-MY2) to the T-LC.

## Conflict of interest

The authors declare that the research was conducted in the absence of any commercial or financial relationships that could be construed as a potential conflict of interest.

## Publisher’s note

All claims expressed in this article are solely those of the authors and do not necessarily represent those of their affiliated organizations, or those of the publisher, the editors and the reviewers. Any product that may be evaluated in this article, or claim that may be made by its manufacturer, is not guaranteed or endorsed by the publisher.

## Footnotes

^1^More than 90% of Chinese idiomatic expressions are quadrisyllabic (Xu, 2006). Zhou (2004) analyzed 17,934 idioms compiled in *Dictionary of Chinese Idioms* and found that 95.57% of idioms collected in the volume are quadrisyllabic.

^2^The L2 of native Mandarin participants is English. Some of them also have a basic knowledge in Japanese. The native languages of participants in the L2 group include English (6), Spanish (6), French (3), Dutch (1), Swedish/Dutch (1), Hungarian (1), and Italian (1). The number in the parenthesis indicates the number of native speakers of a certain language.

^3^Su (2002), Tsou (2012), and Liu et al. (2019) point out that Chinese idiomatic constructions are characterized by syntactic parallelism in that the slots substituted in the constructions are generally synonyms (e.g., [*qian-cun-wan-luo*] 千村萬落: [1 k-village-10 k-hamlet] ‘thousands of villages and hamlets’), antonyms (e.g., [*yi-chang-yi-duan*] 一長一短: ‘merits and demerits’) or semi-bound morphemes (e.g., [*qian-hu-wan-huan*] (千呼萬喚: [1 k-call-10 k-shout] ‘after repeated calls’). The slots inserted in pseudo-idiomatic constructions are neither synonyms nor antonyms, and thus violate the syntactic constraints of Chinese idiomatic constructions.

## Appendix

Idiomatic constructions used for targets.

**Table:**

High frequency idiomatic constructions		Low frequency idiomatic constructions	
千變萬化 Qian-bian-wan-hua	Constant permutations	千仇萬恨 Qian-chou-wan-hen	Deep hatred
千辛萬苦 Qian-xin-wan-ku	Much hardship	千歡萬喜 Qian-huan-wan-xi	Extremely happy
千年萬載 Qian-nian-wan-zai	A very long time	千刀萬剁 Qian-dao-wan-duo	A 1,000 cuts and myriad pieces
千軍萬馬 Qian-jun-wan-ma	A huge army	千變萬狀 Qian-bian-wan-zhuang	Have much variety
千山萬水 Qian-shan-wan-shui	A long and arduous journey	千端萬緒 Qian-duan-wan-xu	With many thoughts in mind
千叮萬囑 Qian-ding-wan-zhu	Exhort repeatedly	千轉萬變 Qian-zhuan-wan-bian	Constant permutations
千差萬別 Qian-cha-wan-bie	Completely different	千嬌萬態 Qian-jiao-wan-tai	Beautiful appearance and figure
千家萬戶 Qian-jia-wan-hu	Every family	千村萬落 Qian-cun-wan-luo	Many villages
千難萬險 Qian-nan-wan-xian	Many hazards and difficulties	千依萬順 Qian-yi-wan-shun	Always obedient
千真萬確 Qian-zhen-wan-que	Absolutely true	千變萬態 Qian-bian-wan-tai	Constant permutations
千恩萬謝 Qian-en-wan-xie	Thank again and again	千思萬慮 Qian-si-wan-lü	Think or consider repeatedly
千態萬狀 Qian-tai-wan-zhuang	Have much variety	千支萬派 Qian-zhi-wan-pai	Many (philosophical, martial, etc.) sects
千峰萬壑 Qian-feng-wan-huo	Many mountains and valleys	千兵萬馬 Qian-bing-wan-ma	A huge army
千秋萬世 Qian-qiu-wan-shi	A long, long time	千回萬轉 Qian-hui-wan-zhuan	Go through ups and downs
千門萬戶 Qian-men-wan-hu	A big house or lots of inhabitants	千章萬句 Qian-zhang-wan-ju	Many phrases and articles
千呼萬喚 Qian-hu-wan-huan	Call repeatedly	千倉萬箱 Qian-cang-wan-xiang	Massive storage of food
千山萬壑 Qian-shan-wan-huo	Many mountains and valleys	千推萬阻 Qian-tui-wan-zu	Do everything to decline
千條萬縷 Qian-tiao-wan-lü	Many threads	千緒萬端 Qian-xu-wan-duan	Tangled thoughts
千乘萬騎 Qian-sheng-wan-ji	Lots of carriages and cavalry	千生萬死 Qian-sheng-wan-si	Very dangerous situation
千言萬語 Qian-yan-wan-yu	Have many words to say	千齡萬代 Qian-ling-wan-dai	Generation after generation
千刀萬剮 Qian-dao-wan-gua	A 1,000 cuts and myriad pieces	千匯萬狀 Qian-hui-wan-zhuang	Have much variety
千頭萬緒 Qian-tou-wan-xu	So many thoughts in one’s mind	千言萬說 Qian-yan-wan-shuo	Have many words to say
千絲萬縷 Qian-tiao-wan-lü	Tangled connections	千妥萬當 Qian-tuo-wang-dang	Very appropriate
千秋萬歲 Qian-qiu-wan-sui	A long, long time	千岩萬穀 Qian-yan-wan-gu	A group of mountain ranges
